# Supplementary material for: Towards decoding individual words from non-invasive brain recordings
Source: Nat Commun. 2025 Nov 26;16:10521. doi: 10.1038/s41467-025-65499-0 (PMC12658044; doi:10.1038/s41467-025-65499-0)
Supplement: Supplementary file 1 — Supplementary Information [file 41467_2025_65499_MOESM1_ESM.pdf]

# Supplementary information

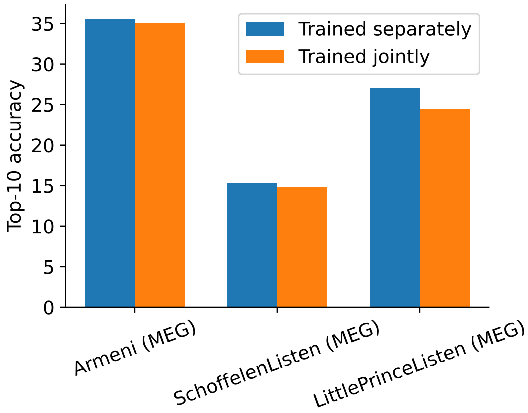


Supplementary Figure 1: **Training jointly versus separately.**
We compare the decoding performance achieved on three MEG listening datasets when training separately on each of them (blue) and training jointly on all of them combined (orange). We selected these three datasets, which all correspond to listening tasks with MEG recordings, in order to minimize the inter-dataset variability. Yet, even in this setting, we do not observe any mutual benefit from training jointly.

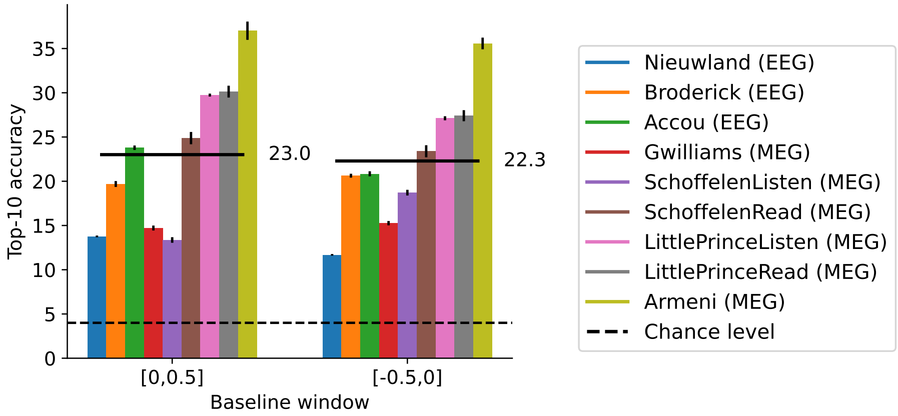


Supplementary Figure 2: **Impact of baselining before or after the stimulus.**
We compare the decoding performance achieved when computing the baseline inside the stimulus window (left) to before the stimulus window (right). Surprisingly, decoding results are slightly better when computing the baseline inside the window, contrary to the traditional approach. The error bars represent the SEM across subjects (the number of subjects for each dataset is reported in table 1).

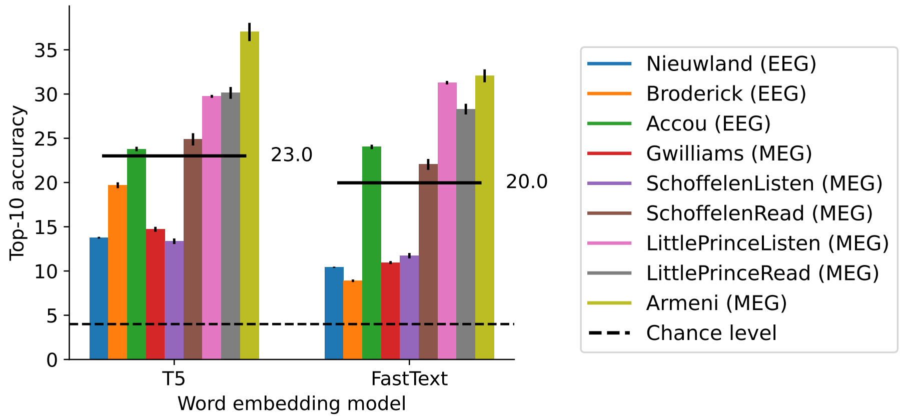


Supplementary Figure 3: **Impact of the word embedding model.**
We compare the decoding performance achieved with the word embeddings obtained with the T5-large model (left) to those obtained with the `FastText` package for word embeddings (right) [75], showing that there is a small improvement when using T5. FastText word embeddings are constructed by representing each word as a bag of character n-grams, which are then used to learn a weighted sum of these character sequences. The model is trained on a large corpus of text using a variant of the skip-gram model, with the goal of predicting a target word given its context words. FastText incorporates subword information by combining character n-grams and the word itself, allowing it to handle out-of-vocabulary (OOV) words by generating embeddings from their character n-grams.
The error bars represent the SEM across subjects (the number of subjects for each dataset is reported in table 1).

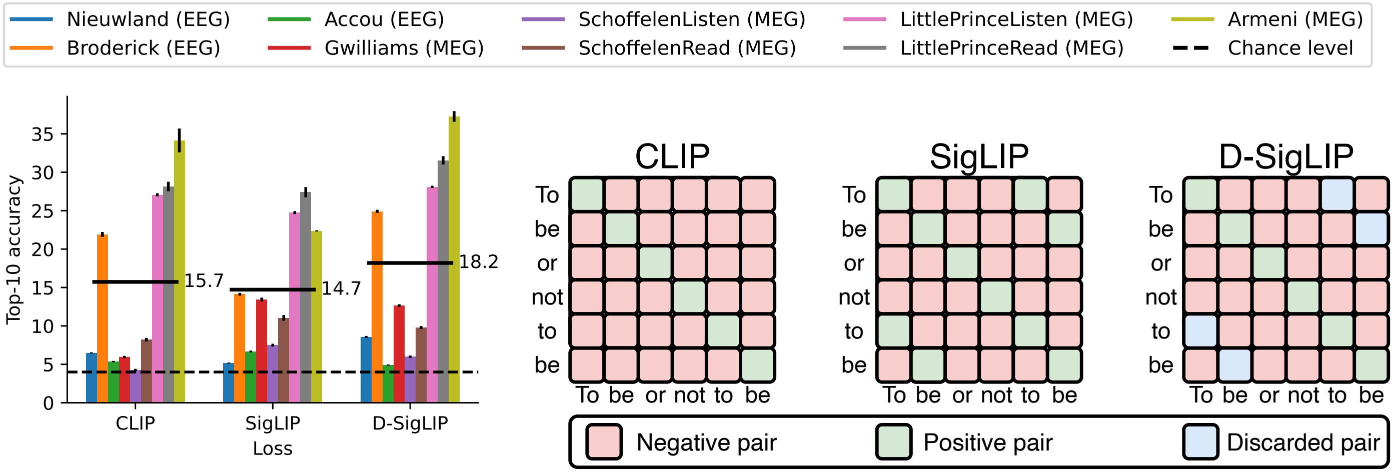


Supplementary Figure 4: **Impact of the loss function.**
We report the average top-10 accuracy on the most frequent 250 words across the participants of all datasets, as well as the SEM. Our D-SigLIP loss significantly outperforms the other ones ($p < 0.005$).
The error bars represent the SEM across subjects (the number of subjects for each dataset is reported in table 1).

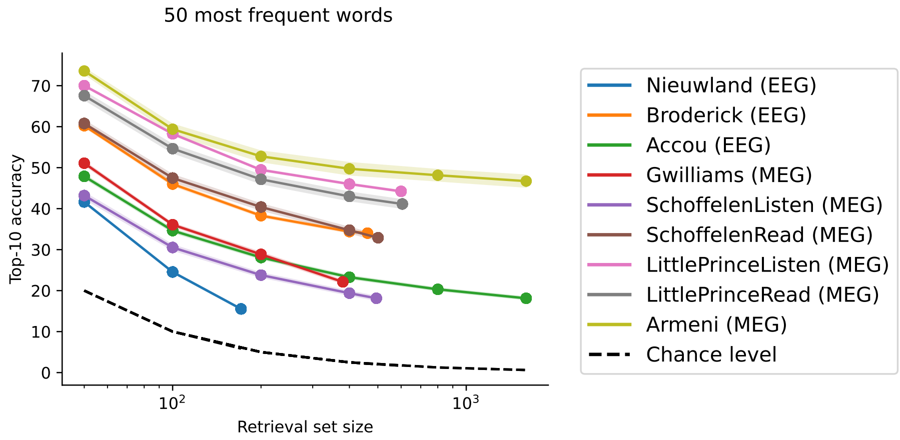


Supplementary Figure 5: **Impact of retrieval set size.**
Decoding performance on the 50 most frequent words as we increase the size of the retrieval set from 50 to 1600 words. Accuracy naturally decreases as the size of the retrieval set increases, but remains well above chance.

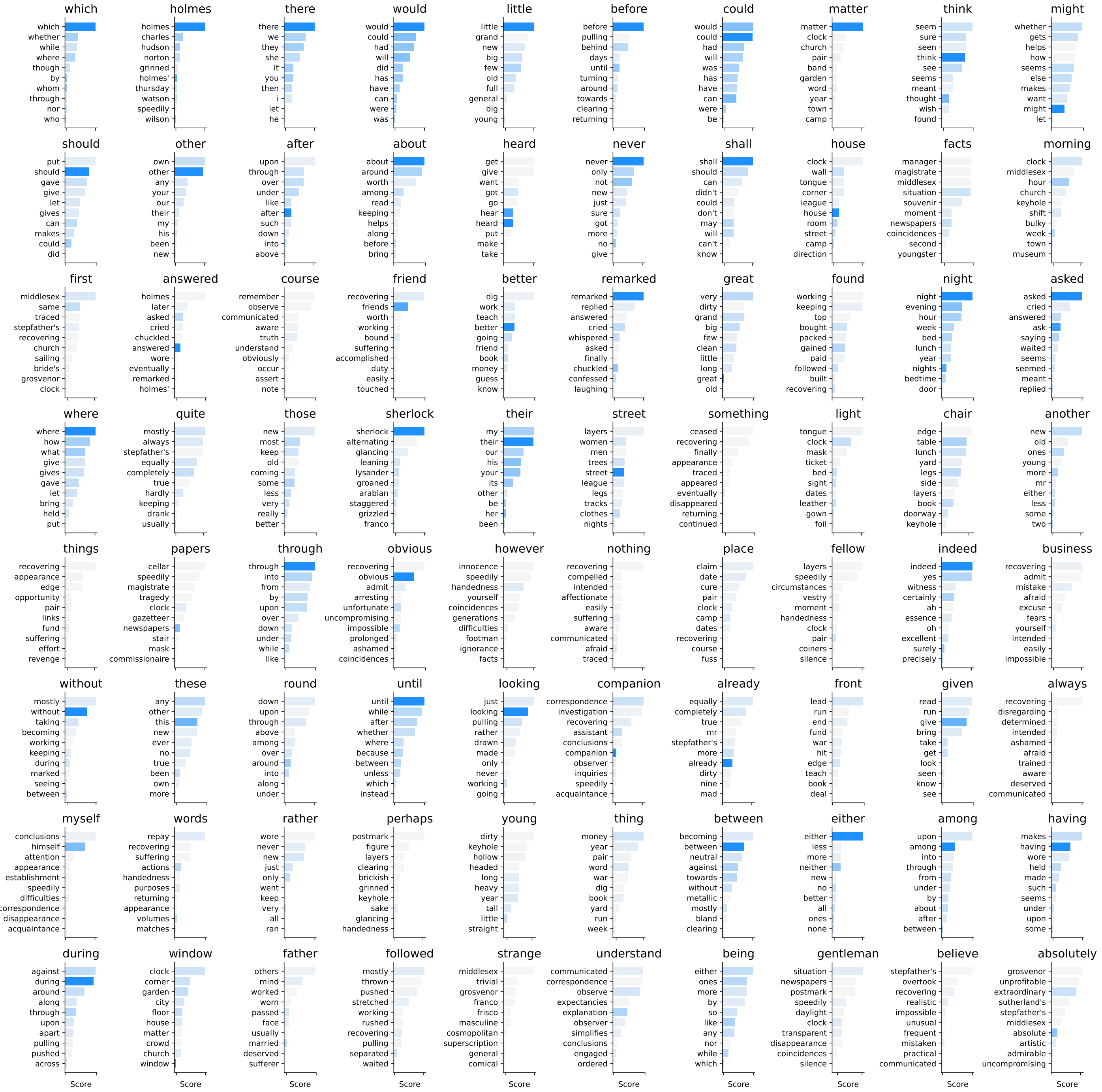

Supplementary Figure 6: **Top predictions on a subset of words.**
We selected the most 80 most frequent words from the test of Armeni *et al.* [26] containing at least five letters.
