# Supplementary material for: Towards decoding individual words from non-invasive brain recordings
Source: Nat Commun. 2025 Nov 26;16:10521. doi: 10.1038/s41467-025-65499-0 (PMC12658044; doi:10.1038/s41467-025-65499-0)
Supplement: Supplementary file 2 — Description of Additional Supplementary Files [file 41467_2025_65499_MOESM2_ESM.pdf]

Description of Additional Supplementary Files

File name : Supplementary Code 1

Description : Decoding individual words from non-invasive brain recordings

This repository supports the paper "Decoding individual words from non-invasive brain recordings". It loads some EEG or MEG datasets and trains a ConvNet, and optionally a Transformer with `use_transformer=True`, to perform word retrieval. See more details in the paper.

****1. Install neuralset and neuraltrain****
The code used here depends on two packages.
The first, called neuraltrain, contains the utilies to train deep learning models. See [`neuraltrain`](neuraltrain/README.md) for installation instructions.
The second, called neuralset, is used for the loading of data from multiple datasets in the literature and will be released publicly later this year.

****2. [Optional] Set up Weights & Biases****
[General instructions](https://docs.wandb.ai/quickstart)

****3. Download the data for the studies of interest****
See `neuralset/studies` for the list of currently supported datasets, and instructions on where to download them from.

****4. Set the path to data and save directory****
Set 3 variables which will determine where the data is stored, where you want to save your results, and the name of the Slurm partition to use.
There are two ways to do this: either change the values in `grids/defaults.py`, either directly set the corresponding environment variables (SAVEPATH, DATAPATH and SLURM_PARTITION).

****5. Run local test (useful for debugging)****
python -m sentence_decoding.grids.test

****6. Run over all datasets on Slurm****
python -m sentence_decoding.grids.final

****7. Monitor training and inspect results****
Head over to *Weights & Biases* to monitor training and inspect results.
